# Metabolic differentiation and intercellular nurturing underpin bacterial endospore formation

**DOI:** 10.1126/sciadv.abd6385

**Published:** 2021-01-22

**Authors:** Eammon P. Riley, Javier Lopez-Garrido, Joseph Sugie, Roland B. Liu, Kit Pogliano

**Affiliations:** 1 Division of Biological Sciences, University of California San Diego, La Jolla, CA, USA.; 2 Max Planck Institute for Evolutionary Biology, Plön, Germany.

## Abstract

Despite intensive research, the role of metabolism in bacterial sporulation remains poorly understood. Here, we demonstrate that *Bacillus subtilis* sporulation entails a marked metabolic differentiation of the two cells comprising the sporangium: the forespore, which becomes the dormant spore, and the mother cell, which dies as sporulation completes. Our data provide evidence that metabolic precursor biosynthesis becomes restricted to the mother cell and that the forespore becomes reliant on mother cell–derived metabolites for protein synthesis. We further show that arginine is trafficked between the two cells and that proposed proteinaceous channels mediate small-molecule intercellular transport. Thus, sporulation entails the profound metabolic reprogramming of the forespore, which is depleted of key metabolic enzymes and must import metabolites from the mother cell. Together, our results provide a bacterial example analogous to progeny nurturing.

## Teaser

Sporulation entails intercellular metabolic dependency and nutrient exchange, providing a bacterial case of progeny nurturing.

## INTRODUCTION

**Fig. 1:**
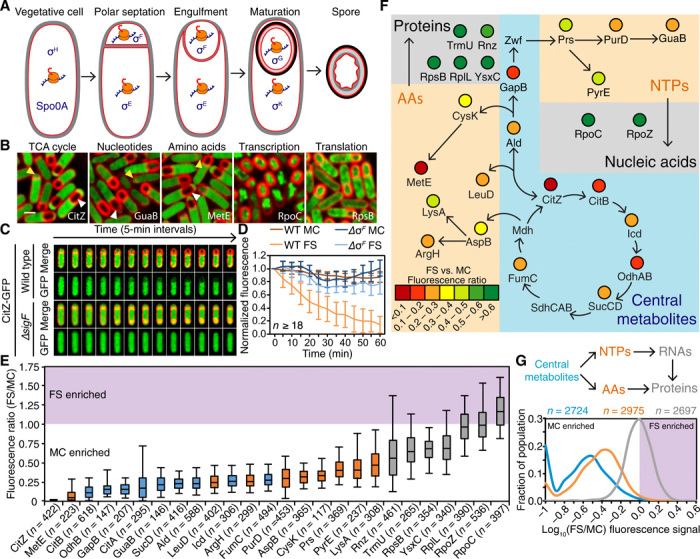
Metabolic reprogramming during sporulation. (**A**) Sporulation pathway in *B. subtilis*. Membranes, red; spore integuments, gray and black. Transcription factors active at each stage are indicated in blue, and active protein synthesis is indicated by orange ribosomes. (**B**) Visualization of GFP fusions (green) to representative metabolic proteins. Membranes are in red. Yellow arrowheads denote sporangia that have just completed polar septation. White arrowheads mark sporangia that have completed engulfment. Scale bar, 1 μm. See also fig. S1A. (**C**) Time-lapse fluorescence microscopy of a sporangium producing CitZ-GFP (green) in wild-type background and in a mutant lacking the forespore-specific transcription factor σ^F^ (Δ*sigF*). Membranes are in red. See also movies S1 and S2. (**D**) CitZ-GFP fluorescence signal in the mother cell (MC; darker shade) and in the forespore (FS; lighter shade) produced in wild-type (orange) or ∆*sigF* sporangia (blue) over time, relativized to the initial signal at *t*_0_. Values represent the mean ± SD. (**E**) Box and whisker plots showing the ratios of the mean forespore fluorescent signal to the mean mother cell fluorescence signal for metabolic enzymes functioning in different metabolic pathways. The fluorescent signals of the different fusions are normalized to cell volume and therefore represent the concentration of the different fusions in each cell. The boxes represent the middle 75% of the data, and the vertical bars represent the middle 90%. Median values are indicated by the horizontal lines inside the boxes. (**F**) Results from (E) superimposed on a metabolic map. AAs, xxxx; NTPs, xxxxx. (**G**) Histogram of forespore–to–mother cell fluorescence ratios of the GFP fusions measured in (E), grouped by tier in the metabolic hierarchy (blue, central metabolism; orange, precursor synthesis; gray, final product assembly). AAs, amino acids; NTPs, nucleoside triphosphates.

Some bacterial species can alternate between a vegetative reproductive cycle in which a single cell produces two metabolically active progeny cells and a sporulation cycle that leads to the formation of a metabolically dormant and extremely resilient endospore (henceforth spore). The sporulation pathway is conserved among different endospore-forming bacteria but is best characterized in *Bacillus subtilis* (*1*, *2*). Sporulation begins with an asymmetric cell division event (polar septation) that gives rise to a sporangium consisting of two cells with different sizes: the smaller forespore and the larger mother cell (Fig. 1A). Following polar septation, the mother cell engulfs the forespore, which becomes enclosed within the mother cell cytoplasm and matures protected from the extracellular medium. Once maturation is complete, the mother cell lyses, thereby releasing the resilient spore into the environment, where it remains dormant until conditions are favorable for germination and the resumption of vegetative growth.

Sporulation involves the differential activation of genetic programs in the mother cell and the forespore, which govern the synthesis of unique sets of proteins in each cell, even throughout late stages of sporulation (*1*, *3*, *4*). The different genetic programs rely upon the sequential activation of cell-specific transcription factors, called σ factors (Fig. 1A), which guide the RNA polymerase to different classes of developmental promoters, driving the expression of sporulation-specific structural genes in a spatiotemporally regulated manner. While the sporulation field has made substantial advances toward deciphering the regulatory mechanisms that trigger the activation of sporulation genetic programs (*1*, *3*, *4*) and the function of sporulation-specific structural proteins (*2*), it remains unclear which metabolic pathways contribute to spore formation and how those pathways are coordinated between mother cell and forespore.

It has long been proposed that sporulation entails an intimate metabolic relationship between the mother cell and the forespore in which the mother cell nurtures the forespore as it transitions to dormancy, providing the metabolic intermediates necessary to support forespore macromolecular synthesis. This is an appealing model because, after engulfment, the forespore loses access to extracellular metabolites and because, immediately after polar septation, the volume of the forespore is just 10% that of the mother cell (*5*) and therefore has relatively few metabolic resources that can be recycled to support forespore assembly. This model is supported by the existence of a sporulation-specific complex consisting of eight mother cell proteins encoded by the *spoIIIA* operon (SpoIIIAA-AH; henceforth A) and the forespore protein SpoIIQ (henceforth Q), which are proposed to form multimeric channels connecting the mother cell and forespore (*6*–*14*) and are required for late forespore gene expression (*7*). These observations have led to the proposal that the A-Q complex provides a gap junction–like feeding tube through which the mother cell nurtures the developing spore by providing small molecules needed for biosynthetic activity (*7*). Recent structural studies support the notion that the A proteins assemble a channel (*9*–*11*, *13*), but transport of small molecules through A-Q channels has not been directly shown. It also remains unclear if the forespore depends on mother cell metabolic capabilities and if the two cells become metabolically differentiated during development. Here, we investigate the metabolic transformations and interactions that accompany spore formation in *B. subtilis*.

## RESULTS

### The mother cell and forespore become metabolically differentiated during sporulation

To gain insights into the metabolic relationship between the mother cell and forespore, we first evaluated if enzymes functioning in different metabolic pathways are enriched in one cell compared to the other. For this purpose, we GFP (green fluorescent protein)–tagged key metabolic enzymes functioning in central carbon metabolism, the synthesis of amino acids and nucleotides, and the assembly of macromolecules (RNA and proteins) and quantified the fluorescence signals in the forespore and the mother cell during development (Fig. 1, B to G, and fig. S1A). We found unexpected differences between the fluorescence signal in the forespore and in the mother cell, which varied in magnitude depending on the pathway (Fig. 1, E to G): Enzymes belonging to central carbon metabolism exhibited a ~4- to >100-fold reduction in the forespore compared to the mother cell; amino acid and nucleotide biosynthetic proteins exhibited forespore reductions ranging from 2- to 12-fold; however, with the sole exception of σ^A^ (fig. S1, B and C; see Supplementary Text), proteins directly involved in transcription and translation were present at similar levels in the mother cell and the forespore, consistent with active RNA and protein synthesis occurring in both cells throughout sporulation.

The reduced forespore levels of proteins involved in central carbon metabolism and metabolic precursor synthesis seemed to be due to the specific disappearance of proteins from the forespore rather than increased synthesis in the mother cell, because the proteins were present in vegetative cells before polar septation, and similar fluorescence signals were observed in the mother cell and the forespore shortly after polar septation (Fig. 1B and fig. S1A). Time-lapse microscopy further supported this point, revealing that a CitZ-GFP fusion protein was rapidly and specifically depleted from the forespore to background levels, despite initially being present at high levels in both the mother cell and forespore immediately following polar septation (Fig. 1, C and D; fig. S1D; and movie S1). Depletion of CitZ-GFP depends on the forespore transcription factor σ^F^ (Fig. 1, C and D; fig. S1D; and movie S2), demonstrating that this is an active, genetically encoded process under sporulation control. In addition, we found no apparent correlation between forespore size and the degree of metabolic asymmetry, suggesting that metabolic enzymes are not cleared from the forespore to generate space to accommodate newly synthesized spore components (fig. S1E). These results suggest that the forespore and mother cell become metabolically differentiated after polar septation. While both cells retain proteins directly involved in transcription and translation, enzymes involved in central metabolism, and amino acid and nucleotide biosynthesis are quickly depleted in the forespore.

### Differential requirement of metabolic pathways in the mother cell and the forespore

Together, the observations above suggest a drastic reduction in forespore metabolic capabilities during engulfment, providing a metabolic basis for forespore nurturing in which the mother cell bears the burden of metabolic precursor biosynthesis to support macromolecular synthesis in both the mother cell and the forespore. For this metabolic nurturing model to be true, we reasoned that two premises should be fulfilled. First, enzymes involved in the synthesis of metabolic precursors should be required preferentially in the mother cell, while those involved in the assembly of macromolecules should be independently required in each cell. Although the asymmetric distribution of metabolic enzymes among the mother cell and the forespore is consistent with this premise, it remains possible that the forespore simply has a lower requirement for metabolic precursors. Second, the assembly of macromolecules in the forespore should depend on mother cell–derived metabolic precursors that are transported directly into the forespore.

**Fig. 2:**
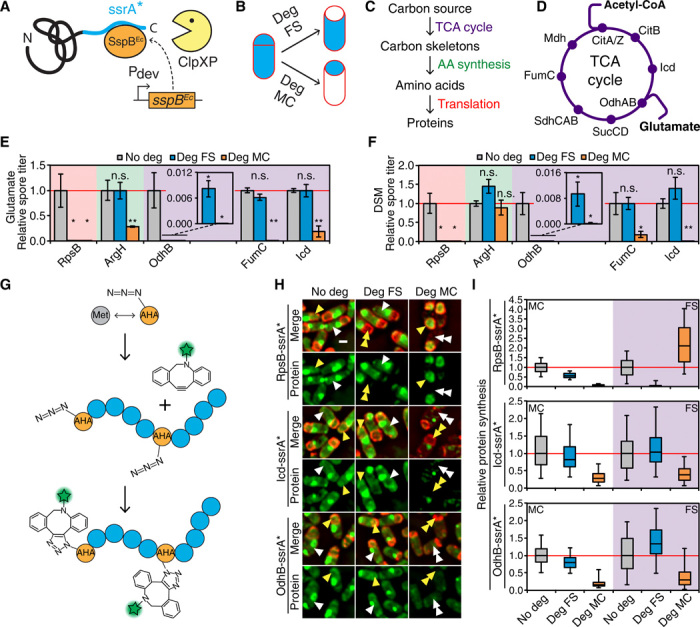
Intercellular metabolic dependency during sporulation. (**A** and **B**) STRP diagram. (**C**) Rationale for identifying cell-specific requirement of metabolic pathways. (**D**) TCA cycle diagram. Different carbon inputs in bold. (**E** and **F**) Heat-resistant spore titers of strains producing different ssrA*-tagged proteins after sporulation in a glutamate defined medium (E) or in a complex medium (DSM) (F). No degradation (No deg), gray; degradation in the forespore (Deg FS), cyan; degradation in the mother cell (Deg MC), orange. Titers of the nondegradation strains normalized to one. Data represent mean ± SEM of at least three experiments. Insets: Zoom of spore titers after OdhB-ssrA* degradation. Statistical significance (unpaired *t* test): n.s., not significant; *P ≤ 0.05; **P ≤ 0.01. (**G**) BONCAT diagram. (**H**) Images of sporangia producing RpsB-ssrA*, Icd-ssrA*, or OdhB-ssrA*, with BONCAT-labeled newly synthesized proteins (green). Membranes are red. White and yellow arrowheads, representative forespores and mother cells, respectively. Single and double arrowheads, high and low protein synthesis levels, respectively. Scale bar, 1 μm. (**I**) Protein synthesis in the mother cell (MC; white shading) and in the forespore (FS; purple shading) in RpsB-ssrA*, Icd-ssrA*, or OdhB-ssrA* strains. No degradation, gray; degradation in the forespore, cyan; degradation in the mother cell, orange. At least 142 sporangia quantified for each strain. Values normalized to the median of tagged-only strain (red line). See Fig. 1E for description of box and whisker plot.

To evaluate the first premise, we took advantage of a recently developed technique called spatiotemporally regulated proteolysis (STRP), which allows the rapid degradation of specific proteins in either the mother cell or the forespore after polar septation (*15*). Briefly, a short degradation tag called ssrA* is fused to the C terminus of target proteins, which are subsequently directed by the SspB^Ec^ adaptor protein to the ClpXP protease for degradation [Fig. 2A; (*16*)]. Cell-specific degradation of target proteins is achieved by expressing *sspB^Ec^* from promoters that are active exclusively in the mother cell or in the forespore after polar septation (Fig. 2B). We focused our efforts on metabolic pathways feeding into protein synthesis (Fig. 2C) by ssrA*-tagging a panel of tricarboxylic acid (TCA) cycle enzymes (OdhB, SucD, FumC, Mdh, CitZ, CitB, and Icd), amino acid biosynthetic enzymes (ArgH, ThrB, and IlvD), and ribosomal proteins (RpsB and RplL). First, we confirmed that the tag did not interfere with protein functionality and that tagged proteins were efficiently degraded upon expression of *sspB^Ec^* (fig. S2). Then, we assessed the requirement for each protein in the mother cell or in the forespore by taking advantage of a unique property of mature spores, namely, their resistance to high temperatures (of over 80°C). We determined the heat-resistant spore titers of strains harboring the different ssrA*-tagged proteins and after degradation of each target protein in either the mother cell or the forespore. Because the requirement for different target proteins can depend on the specific carbon source (Fig. 2D), we induced sporulation in either a defined medium with glutamate as sole carbon source or in a complex, undefined medium containing a variety of carbon sources [Difco sporulation medium (DSM)].

The results can be summarized as follows (Fig. 2, E and F; fig. S3, C and D; and table S1): (i) As expected, degradation of the ribosomal proteins RpsB or RplL in either cell individually produced marked spore titer reductions in both media, consistent with translation occurring independently in the mother cell and forespore. (ii) Amino acid biosynthetic proteins yielded mild spore titer reductions (about two- to threefold) when degraded in the mother cell in the glutamate defined medium. No spore titer defects were observed when degradation was induced in the forespore, suggesting that those pathways are dispensable in the forespore. (iii) Most TCA cycle enzymes also yielded significant spore titer defects when degraded in the mother cell, which varied in severity depending on the target protein and the medium, although we note that aconitase (CitB) has both enzymatic and regulatory activities and that its RNA binding activity has been shown to be important for sporulation (*17*). Thus, the sporulation defects observed following mother cell degradation of aconitase (fig. S3) might be due to its regulatory role rather than to its enzymatic activity. The strongest defects were caused by Icd degradation in DSM medium (8 × 10^3^–fold reduction) and by OdhB degradation in glutamate medium (>10^5^-fold reduction). Degradation of most TCA cycle enzymes in the forespore did not produce spore titer defects in any medium. The lone exception was OdhB, whose forespore degradation reduced the spore titer ~100-fold in both media. Spore titer reductions, however, were up to 750-fold more severe when OdhB was degraded in the mother cell. In addition, further experiments suggested that the requirement of OdhB in the forespore was unrelated to the production of metabolic precursors for protein synthesis (see below). We further characterized the effects of cell-specific degradation of metabolic proteins using fluorescence and phase-contrast microscopy to directly visualize sporulation progression. The cytological results are fully consistent with those of the spore titers (fig. S3, E and F).

### Forespore protein synthesis depends on mother cell metabolic activity

The second premise that the metabolic nurturing model should fulfill is that forespore macromolecular assembly should depend on metabolic precursors that are synthesized in the mother cell and then subsequently transported to the forespore. To test this premise, we first investigated whether forespore protein synthesis depended on mother cell metabolism. To this end, we developed a strategy to measure protein synthesis in the mother cell and forespore in strains in which metabolic enzymes were degraded in the mother cell. We quantified bulk protein synthesis in both cells using bio-orthogonal noncanonical amino acid tagging (BONCAT) and fluorescence microscopy (*18*). Briefly, BONCAT involves the use of azide-alkyne click chemistry to fluorescently label cellular proteins (Fig. 2G). Cells are incubated with the l-methionine analog, l-azidohomoalanine (AHA), which is incorporated into newly synthesized proteins by the endogenous translation machinery, resulting in a general labeling of all newly synthesized proteins. We used BONCAT in conjunction with STRP to measure new protein synthesis in situ in strains in which different proteins were degraded in either the mother cell or the forespore.

We first validated this approach by degrading RpsB, the ribosomal protein S2, to induce a cell-specific translation arrest (Fig. 2, H and I, top, and figs. S4, S5, and S10, C and D). Because a substantial fraction of the new proteins synthesized in the mother cell are coat proteins, which localize around the forespore and complicate the measurement of the fluorescence signals in both cells, we performed all BONCAT experiments in a SpoIVA^−^ background to prevent the attachment of the coat to the forespore (fig. S5). In this background, coat proteins are not attached to the forespore surface and instead assemble into aggregates in the mother cell cytoplasm. Disabling translation in either cell individually produced a rapid, cell-specific inhibition of protein synthesis, illustrating the potential of STRP and BONCAT to dissect the metabolic interactions between cell types during sporulation. We then focused on the TCA cycle, as blocking this process should produce a generalized metabolic shutdown, affecting many downstream pathways. Specifically, we chose two enzymes whose cell-specific degradation produced strong sporulation defects: Icd, which caused sporulation defects only when degraded in the mother cell, and OdhB, which also impaired sporulation, albeit to a lesser degree, when degraded in the forespore. As shown in Fig. 2 (H and I, middle and bottom), strains producing Icd-ssrA* or OdhB-ssrA* exhibited robust protein synthesis in both cells. When either protein was targeted for degradation in the forespore, protein synthesis levels in both the mother cell and forespore were similar to those observed in the absence of degradation, suggesting that TCA cycle activity in the forespore is not required to support protein synthesis in either cell. This result suggests that the sporulation defect observed when OdhB is degraded in the forespore is not due to a protein synthesis defect, and we speculate that it might be related to the maintenance of redox balance through the reduction of NAD^+^ (nicotinamide adenine dinucleotide) to NADH (reduced form of NAD^+^) and H^+^ or to its role in salvaging the cofactor lipoic acid (*19*). Degradation of Icd-ssrA* or OdhB-ssrA* in the mother cell, however, nearly completely abolished protein synthesis in both the mother cell and the forespore, demonstrating that both proteins are required only in the mother cell to support protein synthesis in the two cells. Together, these data demonstrate that shortly after polar septation, forespore protein synthesis becomes dependent on mother cell central carbon metabolism, and suggest that the mother cell supplies the forespore with the metabolic building blocks required for protein synthesis.

### Metabolic building blocks are trafficked between mother cell and forespore

**Fig. 3:**
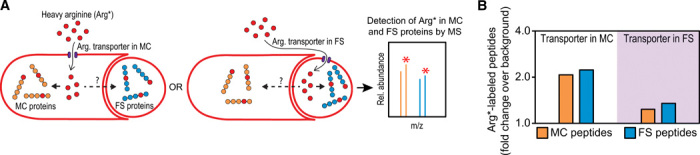
Arginine is transported between the mother cell and the forespore. (**A**) Diagram of the cell-specific SILAC approach used to assess intercellular transport of arginine. See main text for details. (**B**) Fraction of mother cell (MC) peptides (orange) and forespore (FS) peptides (blue) containing heavy arginine (Arg*) when the arginine transporter ArtPQR is produced in the mother cell (from *spoIIM* promoter, white shading) or forespore (from *spoIIQ* promoter, purple shading) compared to the background incorporation obtained when no arginine transporter is produced. More than 3000 mother cell and forespore peptides were analyzed for every strain.

The BONCAT results presented also provide evidence that AHA is trafficked from the mother cell to the forespore, as most sporangia had already completed engulfment at the time point in which AHA was added. Despite losing access to the culture medium, AHA incorporation was still observed in forespore proteins, suggesting that AHA imported by the mother cell is subsequently transported to the forespore. To directly test whether the mother cell provides the forespore with substrates for protein synthesis, we devised a strategy to monitor the transport of amino acids between the two cells. The strategy is based on the stable isotope labeling with amino acids in cell culture (SILAC) technique (*20*) in which cells are incubated with amino acids labeled with heavy, stable isotopes of carbon (^13^C) and nitrogen (^15^N), whose incorporation into proteins can be detected by mass spectrometry (MS). Specifically, we engineered a cell-specific SILAC system in which labeled amino acids are imported by either the mother cell or the forespore (Fig. 3A). To achieve this, we expressed individual amino acid transporters from promoters that are active in only one of the cells, in strains genetically engineered to be otherwise unable to import that specific amino acid. This strategy, we reasoned, would restrict the uptake of the amino acid to the cell type expressing the transporter, leading to incorporation of the label into proteins produced only in that cell. If the amino acid is trafficked between the two cells, however, we would also observe incorporation into proteins produced in the cell not expressing the transporter (Fig. 3A). The mother cell and forespore synthesize unique sets of abundant sporulation proteins that are not found in nonsporulating cells, so one can readily distinguish proteins produced by each cell using MS and detect the incorporation of labeled amino acids after transfer from one cell to the other.

Our STRP results implicate arginine as a likely candidate for intercellular transport (Fig. 2), so we focused our efforts on arginine importers. To develop a cell-specific arginine labeling system (fig. S6), we constructed strains lacking all putative arginine permeases and in which the expression of *artPQR*, which encodes a high-affinity arginine ABC (ATP-binding cassette) transporter (*21*, *22*), is under the control of mother cell– or forespore-specific promoters. To maximize the incorporation of exogenous arginine into sporulation proteins, we used strains in which arginine synthesis was blocked in both the mother cell and the forespore by STRP-mediated degradation of ArgH-ssrA* (fig. S6). This framework allows the cell-specific uptake of arginine during sporulation, but not during growth (figs. S7 and S8). As a control, we used a strain lacking all predicted arginine transporters that does not express *artPQR* from sporulation promoters, which shows reduced ability to import arginine during both growth and sporulation (figs. S7 and S8). We incubated sporulating cells of the control strain and of strains producing ArtPQR in either cell with modest (0.1 mM) concentrations of “heavy” arginine in which all the carbon and nitrogen atoms are replaced by their respective stable heavy isotopes, ^13^C and ^15^N. We prepared protein extracts of spores produced by the different strains, digested them with trypsin, and performed MS to detect the incorporation of heavy arginine in mother cell– and forespore-specific peptides.

For every strain, we detected more than 3000 sporulation-specific peptides that contained at least one arginine residue and classified them into mother cell– and forespore-specific peptides [based on (*23*)] to determine the percentage of heavy arginine incorporation in proteins from either cell (data S1). In the control strain, which lacks all predicted arginine transporters, heavy arginine was incorporated into ~12% of the sporulation-specific peptides (fig. S9). This background incorporation is likely due to nonspecific arginine transport, as arginine auxotrophic strains lacking all arginine transporters are still able to grow when high enough concentrations of arginine are supplied (figs. S7 and S8). However, expression of *artPQR* in the mother cell led to a greater than twofold increase in heavy arginine incorporation in mother cell peptides compared to the control strain (Fig. 3B and fig. S9), indicating that the transporter was produced and contributed to arginine uptake by the mother cell. *artPQR* expression in the mother cell also increased heavy arginine incorporation into forespore-specific peptides more than twofold compared to the control. Thus, arginine imported by the mother cell is subsequently incorporated into forespore proteins, which requires transport across the two lipid bilayers separating the two cells.

Expression of *artPQR* in the forespore produced only a slight increase (~1.35-fold over background) in the percentage of forespore peptides containing heavy arginine (Fig. 3B and fig. S9). This might be due to the fact that, after engulfment, the forespore becomes sequestered within the mother cell cytoplasm and loses access to the culture medium, leaving only a short window of opportunity for the forespore to import arginine. In keeping with this hypothesis, heavy arginine was more efficiently incorporated into forespore proteins produced during engulfment than proteins produced after engulfment completion, encoded by genes transcribed by σ^F^ or σ^G^, respectively (fig. S9). Forespore expression of *artPQR* also modestly increased labeling of mother cell proteins (Fig. 3B), predominantly for proteins synthesized during engulfment (fig. S9), suggesting that, in principle, arginine can be transported between the mother cell and the forespore bidirectionally. However, our findings that proteins involved in arginine biosynthesis are only required in the mother cell (Fig. 2) and disappear from the forespore after sporulation initiation (Fig. 1) suggest that arginine is primarily transported from the mother cell to the forespore under normal physiological conditions.

### A-Q complex is required for bidirectional intercellular small-molecule transport

**Fig. 4:**
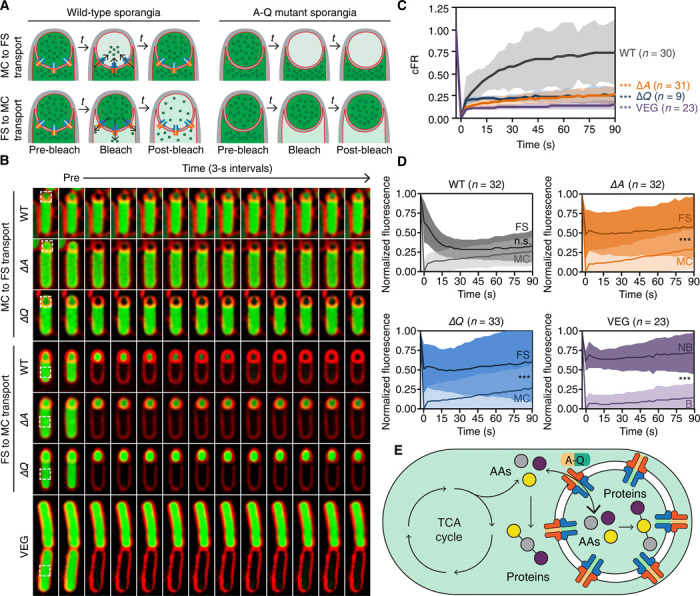
A-Q is required for transport of small molecules between mother cell and forespore. (**A**) Strategy to assay intercellular transport of small molecules using calcein labeling (green) and photobleaching. See main text for details. (**B**) Photobleaching experiments of calcein-loaded wild-type (WT), ∆*A* and ∆*Q* sporangia, or vegetative cells (VEG). Photobleached region indicated by white dotted square. See movies S3 to S9 for additional examples. (**C**) Fluorescence recovery of wild-type (gray), ∆*A* (orange), or ∆*Q* (blue) forespores, or vegetative cells (purple), computed as described in Materials and Methods. The average recoveries of bleached cells are indicated by solid lines, and SDs are indicated by shaded regions for each strain. The number of cells analyzed (*n*) for each strain is indicated to the right of the graph, and unpaired *t* tests were used to determine the statistical significance of the difference between each strain compared to wild type at the final time point (***P ≤ 0.001). (**D**) Fluorescence loss of wild-type (gray), ∆*A* (orange), ∆*Q* (blue) sporangia, or vegetative cells (VEG) (purple), normalized and corrected as described in Materials and Methods. Average fluorescence values of the forespore (dark) and mother cell (light) for each strain are indicated by solid lines, and standard deviations are indicated by shaded regions. The number of cells analyzed (*n*) for each strain is indicated above the respective graph, and unpaired *t* tests were used to determine the statistical significance of the difference between mother cell and forespore fluorescence values for each strain at the final time point (***P ≤ 0.001). NB, not bleached. (**E**) Model of metabolic coupling between mother cell and forespore.

We next evaluated the role played by A-Q channels in mediating metabolic exchange, a role that has been advanced based on the requirement of A-Q for sustained forespore gene expression (*7*). We first used BONCAT to confirm that no forespore protein synthesis is observed in mutants lacking A or Q, despite mother cell protein synthesis continuing at a level similar to wild type (fig. S10, A and B). We next sought to test whether A-Q mediates the transport of small molecules between the mother cell and forespore. We were unable to use the heavy arginine labeling method for this purpose, because mutants lacking A-Q fail to synthesize forespore-specific proteins. We therefore used an alternative strategy (*24*) that entails loading cells with calcein, a fluorescent small molecule that cannot diffuse across membranes, and performing fluorescence recovery after photobleaching (FRAP) to test movement between cells (Fig. 4A).

We loaded sporulating cells with calcein AM, a nonfluorescent, membrane-permeant acetoxymethylester derivative of calcein that can be processed by nonspecific intracellular esterases upon uptake, rendering the calcein both fluorescent and membrane impermeable. This treatment produced uniform fluorescent labeling of vegetative and sporulating cell cytoplasms (Fig. 4B). To validate this approach, we first monitored the fluorescence recovery of vegetative cells separated by complete septa. These cells failed to recover (Fig. 4, B and C; fig. S11A; and movie S3), indicating that calcein is not transported across vegetative septa and that there is no substantial uptake of calcein AM from the medium during the experiment. We next bleached the forespores of engulfing sporangia that were either wild-type or lacking A or Q and monitored fluorescence recovery by microscopy. Bleached forespores of wild-type sporangia exhibited a rapid and significant recovery of fluorescence signal within 90 s (Fig. 4, B and C; fig. S11A; and movie S4), indicating that small molecules can move from the mother cell to the forespore. However, the fluorescent signal did not recover in forespores of sporangia lacking either A or Q (Fig. 4, B and C; fig. S11A; and movies S5 and S6), suggesting that the small-molecule calcein is trafficked from the mother cell to the forespore in an A-Q–dependent manner.

We next tested whether A-Q could mediate calcein transport from the forespore to the mother cell. To do so, we used the same strategy as above, but we photobleached calcein fluorescence in mother cells rather than in forespores (Fig. 4A, bottom). Because the forespore is substantially smaller in volume than the mother cell, transport of fluorescent calcein from the forespore might not produce an obvious increase of fluorescence in the mother cell, so instead we measured fluorescence loss in the forespore after photobleaching the mother cell. In wild-type sporangia, calcein fluorescence was rapidly lost in the forespore after bleaching the mother cell, reaching fluorescence levels not significantly different from those of bleached mother cells within 63 s (Fig. 4, B and D; fig. S11, B and C; and movie S7). In mutants lacking A or Q, however, forespore fluorescence loss was markedly slower and fluorescence levels remained significantly higher than those in the mother cell after 90 s (Fig. 4, B and D; fig. S11, B and C; and movies S8 and S9). This suggests that A-Q channels allow the bidirectional movement of the small-molecule calcein between the mother cell and the forespore. This opens the possibility that A-Q provides the sporangium with a syncytium-like organization in which the concentrations of small molecules equilibrate between the two cells via A-Q channels. Under physiological conditions, however, we presume that the transport of metabolic precursors from mother cell to forespore predominates, because the forespore loses the ability to acquire nutrients from the environment once engulfment completes (see Supplementary Text).

## DISCUSSION

The findings presented in this article demonstrate that sporulation entails a nourishing relationship between two cells that become metabolically differentiated after a single cell division event (Fig. 4E): Following polar septation, the forespore rapidly shuts down central and intermediary metabolism, becoming dependent on the mother cell for metabolic precursors, which are trafficked between the two cells and used for the synthesis of macromolecules. We provide evidence that A-Q functions as a nonspecific metabolic conduit, allowing the mother cell to bear the burden of precursor biosynthesis and to nurture the forespore as it transitions to dormancy. The metabolic reprogramming of the forespore might facilitate the developmental transition to dormancy and establish a metabolic environment conducive to spore revival. The metabolic relationship formed between the mother cell and forespore therefore represents a bacterial example of progeny nurturing, a trait typically associated with higher eukaryotes.

## MATERIALS AND METHODS

### Experimental methods

#### Strain construction

All the strains used in this study are derivatives of *B. subtilis* PY79. A complete list of strains is provided in table S2. The plasmids and oligonucleotides used to construct the different strains are provided in tables S3 and S4, respectively. Detailed descriptions of plasmid construction can be found in Supplementary Text.

Here, we provide a description of the proteins tagged with ssrA* (Fig. 2 and figs. S2 and S3). TCA cycle: CitZ, major citrate synthase (this protein was degraded in a background lacking the minor citrate synthase, CitA); CitB, aconitase; Icd, isocitrate dehydrogenase; OdhB, E2 subunit of 2-oxoglutarate dehydrogenase; SucD, α subunit of succinyl–coenzyme A (CoA) synthetase; FumC; fumarase; Mdh, malate dehydrogenase. The addition of the ssrA* tag to components of the succinate dehydrogenase complex severely affected protein functionality, and they were excluded from this study. Amino acid biosynthetic proteins: ArgH, argininosuccinate lyase, required for arginine synthesis; IlvD, dihydroxy-acid hydratase, required for the synthesis of branched-chain amino acids (leucine, isoleucine, and valine); ThrB, homoserine kinase, required for threonine synthesis. Ribosomal proteins: RpsB, ribosomal protein S2; RplL, ribosomal protein L12.

#### Culture conditions

*B. subtilis* strains were routinely grown on LB plates at 30°C. We induced sporulation in two different ways: (i) by resuspension in A + B sporulation medium containing glutamate as the sole carbon source (*25*), after growing the bacteria in one-fourth diluted LB to OD_600_ (optical density at 600 nm) ~ 0.5 (*26*) (sporulation induction was considered to be the moment in which the cells were resuspended in A + B medium), and (ii) by exhaustion in DSM (*27*), containing beef extract and peptones as carbon and nitrogen sources. In DSM, the cells grow until the nutrients become limiting, and sporulation is induced at the onset of stationary phase, which is characterized by a plateau in the growth curve. Sporulation cultures were grown at 37°C for batch culture experiments and at 30°C for time-lapse microscopy experiments. Arginine auxotrophic strains lacking both ArtPQR and RocC were cultured in LB medium supplemented with a high concentration of l-arginine (10 mM), which allowed enough nonspecific import of l-arginine to support growth.

#### Fluorescence microscopy

Cells were visualized on an Applied Precision DV Elite optical sectioning microscope equipped with a Photometrics CoolSNAP-HQ^2^ camera. Images were deconvolved using SoftWoRx v5.5.1 (Applied Precision). The median focal planes are shown.

##### Batch culture microscopy to follow sporulation progression

We used microscopy to assay for the completion of two developmental milestones: engulfment and forespore maturation (fig. S3F). Engulfment completion was determined using a well-characterized membrane fission assay (*28*). Briefly, cells are treated with a red membrane-impermeable membrane dye, FM 4-64, and a green membrane-permeable membrane dye, MitoTracker Green. During engulfment, forespore membranes are accessible to both the red and green dyes. However, after engulfment, forespore membranes cannot be stained by the red dye and are clearly visible as green ovals inside the mother cells. Forespore maturation was assessed by phase-contrast microscopy. Mature spores contain low water content, which endows them with a bright appearance under phase-contrast microscopy. This allows the extent to which developing spores become phase bright to be used as a proxy for spore maturation.

For batch culture microscopy, cells contained in 12 μl of culture were transferred to 1.2% agarose pads, prepared using A + B medium or one-fourth diluted DSM, either 3 or 6 hours after sporulation induction. Membranes were stained with FM 4-64 (0.5 μg/ml) (Life Technologies) and MitoTracker Green (1 μg/ml) (Life Technologies). FM 4-64 was added to the agarose pad, whereas MitoTracker Green was mixed with the cells before transferring them to the pad.

##### Time-lapse microscopy

For time-lapse microscopy, sporulation was induced at 30°C. To visualize the membranes, FM 4-64 (0.5 μg/ml) was added to the culture 1 hour after sporulation induction and incubated for two additional hours under standard culturing conditions. Seven-microliter samples were taken 3 hours after resuspension and transferred to agarose pads prepared as follows: two-third volume of supernatant from the sporulation culture, one-third volume of 3.6% agarose in fresh A + B sporulation medium, and FM 4-64 (0.17 μg/ml). Pads were partially dried, covered with a glass slide, and sealed with petroleum jelly to avoid dehydration during time-lapse imaging (*29*). Pictures were taken in an environmental chamber at 30°C every 5 min for at least 2 hours. Excitation/emission filters were tetramethyl rhodamine isothiocyanate (TRITC)/CY5 for membrane imaging and fluorescein isothiocyanate (FITC)/FITC for GFP imaging. Excitation light transmission was set to 5% for membrane imaging and 32% for GFP imaging to minimize phototoxicity. Exposure time was 0.1 and 0.3 s for membrane and GFP imaging, respectively. For presentation purposes, sporangia were aligned vertically (with forespore on top) by rotating them using Photoshop.

#### Spore titer assays

Sporulation was induced in 2 ml of DSM or in 10 ml of A + B resuspension medium and was allowed to proceed at 37°C for 24 hours. Two milliliters of culture was then heated at 80°C for 20 min, serially diluted in 1× T-base, plated on LB, and incubated overnight at 30°C. Spore titers were calculated based on colony counts.

#### BONCAT

Sporulation was induced in either A + B medium or DSM, as indicated, at 37°C. One hour after sporulation induction, FM 1-43FX (0.5 μg/ml) (Thermo Fisher Scientific) was added to cultures. AHA (100 μM) (Thermo Fisher Scientific) was added to cultures 3.75 hours following sporulation induction and incubated under standard culturing conditions for 15 min.

Cells were fixed and permeabilized as described previously (*30*). Briefly, 0.5 ml of culture was fixed at room temperature for 20 min in a solution containing 20 mM sodium phosphate (pH 7.4), 2.6% (w/v) paraformaldehyde (EM Sciences), and 0.06% (w/v) glutaraldehyde (EM Sciences). Samples were washed three times with phosphate-buffered saline (PBS) and once with GTE [50 mM glucose, 10 mM EDTA, and 20 mM tris (pH 7.4)] and then resuspended in 200 μl of GTE. Fixed cells were deposited on 15-well glass multitest slides (AP Biomedicals LLC) that were pretreated with 0.1% poly-l-lysine (Sigma-Aldrich) for 5 min and washed twice with sterile water. Fixed cells were permeabilized by adding lysozyme (2 mg/ml) and incubating at room temperature for 4 min. Fixed cells on slides were washed three times with PBS.

Strain-promoted click labeling was performed roughly in accordance with previously described methods (*31*). All of the following incubations were performed in the dark to preserve the fluorescent signal. Fixed cells on multiwell slides were treated with 100 mM 2-chloroacetamide in tris (pH 7.4) and incubated at 37°C for 1 hour to block free thiol functional groups. Following the 1-hour incubation, Alexa Fluor 647 DIBO Alkyne (Thermo Fisher Scientific) was added directly to the 2-chloroacetamide solution to a final concentration of 1 μM and incubated at 37°C for an additional 30 min to promote click labeling. The 2-chloroacetamide dye mixture was then gently removed from the slide by aspiration. Unbound dye was removed through a series of washes, gently aspirating between each step. First, slides were treated with PBS and incubated at 37°C for 10 min. Next, slides were treated with 50% dimethyl sulfoxide in PBS and incubated at room temperature for 20 min. Then, slides were washed three times with PBS, incubating for 3 min at room temperature for each wash. Last, slides were washed and dehydrated with an increasing ethanol series, incubating for 3 min at room temperature for each 50, 80, and 96% ethanol. Slides were air-dried and equilibrated in equilibration buffer (Life Technologies) for 5 min and then washed again with fresh equilibration buffer. Slides were treated with antifade reagent in glycerol/PBS (Life Technologies) and covered with a glass slide.

All BONCAT experiments were performed in a ∆*sigG* and ∆*spoIVA* genetic background. *sigG* encodes the sigma factor responsible for late forespore gene expression. Because some degradation strains fail to complete engulfment efficiently and because early forespore gene expression continues in the absence of SigG (*7*), performing these experiments in a ∆*sigG* background ensured that any observed differences in protein synthesis was not a consequence of the failure to activate late gene expression. To facilitate quantification of mother cell and forespore protein synthesis, all BONCAT experiments were performed in strains lacking SpoIVA to block coat assembly around the forespore (fig. S5). The spore coat consists of more than 70 mother cell–synthesized proteins that encase the forespore and comprise 37% of the total spore protein (*32*). SpoIVA is a protein that constitutes the basement layer of the spore coat and is required for the recruitment and anchoring of coat proteins to the surface of the forespore, but not for the assembly of other coat layers (*33*). Therefore, ∆*spoIVA* mutants form aggregates of coat in the mother cell cytoplasm rather than at the forespore periphery, allowing mother cell protein synthesis to be easily distinguished from forespore protein synthesis.

Mother cell and forespore protein synthesis were quantified in sporangia from three microscopy fields using Fiji software. To determine protein synthesis levels, eight optical sections spanning 1.05 μm from deconvolved images were summed using Fiji’s Z Project tool. Mean Alexa Fluor 647 fluorescence intensities of the forespore and mother cell were determined separately by drawing a polygon encompassing the entire cell and then subtracting the mean background intensity plus 2 SDs. Values for the mother cell and the forespore were made relative to the median fluorescence signal of the respective cell type in an isogenic strain expressing only the –ssrA* tagged protein (for cell-specific inactivation of translation or the TCA cycle) or an isogenic wild-type strain (for Q-A mutants).

#### Fluorescence recovery after photobleaching

Sporulation was induced in A + B medium. Two hours after resuspension, the medium was supplemented with calcein AM (100 μg/ml) (Thermo Fisher Scientific), and the cells were incubated at 37°C for one extra hour. Then, cells were washed three times with 1 ml of fresh A + B medium without calcein AM and placed onto 1.2% agarose pads, supplemented with FM 4-64 to stain membranes. Before performing FRAP, a static membrane picture was taken (excitation/emission, TRITC/CY5; exposure time, 0.15 s) and was used as reference to determine the position of mother cells and forespores. The FRAP experiment was performed as follows: Before the photobleaching event, intracellular fluorescent calcein was imaged using FITC/FITC filters and 0.4-s exposure time. Subsequently, forespore calcein fluorescence was bleached with a 0.1-s pulse from a 488-nm argon laser set to 30% power, and calcein fluorescence images were collected every 3 s for 90 s, using the same filters and exposure time as the prebleach imaging. Quantification was performed as described previously (*34*). Briefly, we calculated the corrected fluorescence recovery (cFR) by determining the relative intensity of the bleached cell to the unbleached cell, and defining the prebleaching ratio as cFR = 1 and the ratio immediately following the bleaching event as cFR = 0. Average cFR curves were obtained by averaging fluorescence recovery values of different bleached cells at the same time points for each strain.

#### Fluorescence loss in photobleaching

Sporulation was induced in A + B medium. Two hours after resuspension, the medium was supplemented with calcein AM (100 μg/ml) (Thermo Fisher Scientific), and the cells were incubated at 37°C for one extra hour. Then, cells were washed three times with 1 ml of fresh A + B medium without calcein AM and placed onto 1.2% agarose pads, supplemented with FM 4-64 to stain membranes. Before performing FRAP, a static membrane picture was taken (excitation/emission, TRITC/CY5; exposure time, 0.15 s) and was used as reference to determine the position of mother cells and forespores. The fluorescence loss in photobleaching (FLIP) experiment was performed as follows: Before the photobleaching event, intracellular fluorescent calcein was imaged using FITC/FITC filters and 0.4-s exposure time. Subsequently, mother cell calcein fluorescence was bleached with a 0.1-s pulse from a 488-nm argon laser set to 70% power, and calcein fluorescence images were collected every 3 s for 90 s, using the same filters and exposure time as the prebleach imaging. A standard curve was generated by measuring the average fluorescence loss of cells distal to the bleaching event (fig. S7D), and this was used to correct for the loss of fluorescence caused by repeated image acquisition rather than intercellular transport (fig. S7E). Fluorescence values were background-subtracted and normalized such that the prebleach values were equal to 1 for both the mother cell and forespore and such that the mother cell values immediately following the bleaching event were equal to zero. Following correction using the standard curve, average normalized fluorescence values for mother cell and forespore were obtained by averaging normalized fluorescence values of different bleached cells at the same time points for each strain.

#### Forespore–to–mother cell GFP fluorescence ratio quantification

To be able to unambiguously distinguish if tagged proteins were present in the forespore or in the mother cell, we excluded membrane-associated proteins from the analysis. The ratio of GFP intensity between forespore and mother cell was measured semiautomatically using a custom script in MATLAB 2017b. Forespores and mother cell objects were identified and segmented from a single FM 4-64 channel by taking advantage of the increased fluorescence around the forespore. Thresholding high allowed identification of forespores, while thresholding low gave the whole cell outline. Subtracting the forespore image from the whole cell image allowed mother cells to be identified. Forespores were then paired to their corresponding mother cells first by filtering mother cell objects based on Euclidean distance and then by the orientation of the mother cell. These filters were made strict to reduce false matches. GFP fluorescence ratio was calculated by using the paired forespore–mother cell objects to mask the GFP fluorescence image to obtain a mean fluorescence intensity for both.

#### Quantification of native CitZ-GFP depletion

Mean GFP fluorescence intensity of the median focal plane was quantified separately for the forespore and mother cell by drawing a polygon encompassing the entire area of each cell type using Fiji. The mean background intensity was subtracted from each measurement. The mean fluorescence intensities for each cell type were averaged, and the initial values were normalized to one.

#### Quantification of phase-bright spores

Measurements of phase-bright spore intensity were obtained in a semiautomated manner from images of sporulating cells at 6 hours after induction using a custom script in MATLAB 2017b, applied to the median focal plane. FM 4-64, which stained the outer cell membranes, provided a fluorescent image to threshold, segment, and count the total number of cells present. The complement of the fluorescent FM 4-64 image provided a mask to segment the background for subsequent subtraction from the phase-bright image. In addition, a second membrane dye, MitoTracker Green, capable of diffusing into the cell, was used to stain fully engulfed forespore membranes. Thus, an image created from the difference in signals of both membrane dyes would only show fully engulfed forespores. Using this new image and filtering out those forespores, which contained objects from concurrent 4′,6-diamidino-2-phenylindole (DAPI) staining (as DAPI is unable to enter fully engulfed spores), ensured that all identified forespores were engulfed. For each forespore identified in this way, the average phase intensity was calculated, with the average background phase intensity removed. A phase intensity threshold of 0.1 arbitrary unit was used to constitute “phase bright.” This threshold was selected because it was the lowest value above which most of the engulfment completed wild-type population was found at 6 hours after sporulation induction. In addition, the populations of known phase-bright mutants failed to reach this threshold.

#### Stable isotope labeling with amino acids in cell culture

Sporulation was induced in A + B medium at 37°C. Cultures were supplemented with 0.1 mM l-arginine-^13^C_6_,^15^N_4_ hydrochloride (Sigma-Aldrich) 2 hours after sporulation induction, and sporulation was allowed to proceed for 72 hours. Formation of mature spores was monitored by phase-contrast microscopy. To achieve arginine uptake by the mother cell, *artPQR* was expressed from *spoIIM* or *spoIVA* promoters. To achieve arginine uptake by the forespore, *artPQR* was expressed from *spoIIQ* or *spoIIR* promoters. To maximize the incorporation of exogenous arginine into sporulation proteins, we used strains in which arginine synthesis was blocked in both the mother cell and the forespore by STRP-mediated degradation of ArgH-ssrA*. In addition, wild-type cells were sporulated in the absence of 0.1 mM l-arginine-^13^C_6_,^15^N_4_ hydrochloride to ensure that labeled peptides were only detected when the medium was supplemented with heavy arginine.

#### Spore purification

Seventy-two–hour sporulation cultures were pelleted and washed once with 4°C sterile water. To lyse remaining vegetative cells, spore samples were incubated overnight at 4°C in sterile water. The next day, the spores were pelleted by centrifugation, washed once with 4°C sterile water, and incubated again overnight at 4°C in sterile water. Spores were then further purified over a phosphate–polyethylene glycol aqueous biphasic gradient as previously described (*35*). The spore-containing organic phase (top) was harvested and washed with 50 or more volumes of 4°C sterile water at least three times. The spores were pelleted, resuspended in fresh sterile water, and stored at 4°C. Sample purity was evaluated using phase-contrast microscopy.

#### Extraction of spore coat proteins

Purified intact spores were diluted to approximately 5 × 10^9^ spores/ml in 0.1 M NaOH and incubated at 4°C for 15 min, vortexing periodically. Samples were pelleted by spinning at 10,000*g* for 10 min at 4°C. The supernatant was applied to a 2-ml 3.5K molecular weight cutoff (MWCO) dialysis cassette (Thermo Fisher Scientific) and dialyzed against 1 liter of 0.5 M sodium acetate/acetic acid buffer (pH 5.0) and then against four changes of 1 liter of deionized water at 4°C over the course of 48 hours. Dialyzed material was harvested and submitted for MS analysis.

#### Extraction of small acid soluble proteins

Purified intact spores were diluted to approximately 5 × 10^9^ spores/ml in 4°C 2 M HNO_3_ and incubated on ice for 30 min. Samples were pelleted by spinning at 10,000*g* for 10 min at 4°C. The supernatant was applied to a 2-ml 3.5K MWCO dialysis cassette (Thermo Fisher Scientific) and dialyzed against five changes of 1 liter of 1% acetic acid at 4°C over the course of 48 hours. Dialyzed material was harvested and submitted for MS analysis.

#### Sample preparation for MS

MS samples were prepared and analyzed by the University of California, San Diego Biomolecular and Proteomics MS Facility (http://bpmsf.ucsd.edu/). An equal volume of 6 M guanidine solution was added to 100 μl of protein extract and mixed. The samples were then boiled for 5 min followed by 5-min cooling at room temperature. The boiling and cooling cycle was repeated a total of three cycles. The proteins were precipitated with addition of methanol to a final volume of 90% followed by vortexing and centrifugation at maximum speed on a benchtop microfuge (14,000 rpm) for 10 min. The soluble fraction was removed by flipping the tube onto an absorbent surface and tapping to remove any liquid. The pellet was resuspended in 200 μl of 8 M urea made in 100 mM tris (pH 8.0). To reduce and alkylate samples, TCEP [tris (2-carboxyethyl) phosphine] was added to a final concentration of 10 mM and chloroacetamide was added to a final concentration of 40 mM and vortexed for 5 min. Three volumes of 50 mM tris (pH 8.0) were added to the sample to reduce the final urea concentration to 2 M. Samples were digested with trypsin (trypsin:protein ratio of 1:50) and incubated at 37°C for 12 hours. The solution was then acidified using trifluoroacetic acid (TFA) (0.5% TFA final concentration) and mixed. Samples were desalted using 100 mg of C18-StageTips (Thermo Fisher Scientific) as described by the manufacturer protocol. Samples were resuspended in sample loading buffer, and the peptide concentration was measured using bicinchoninic acid (BCA) (Thermo Fisher Scientific). A total of 1 μg was injected for each label-free quantification run.

#### LC-MS/MS analysis

Trypsin-digested peptides were analyzed by ultrahigh-pressure liquid chromatography (UPLC) coupled with MS/MS (LC-MS/MS) using nanospray ionization. The nanospray ionization experiments were performed using an Orbitrap Fusion Lumos Hybrid mass spectrometer (Thermo Fisher Scientific) interfaced with nanoscale reversed-phase UPLC (Thermo Dionex UltiMate 3000 RSLC Nano System) using a 25-cm, 75-μm ID (internal dimensions) glass capillary packed with 1.7-μm C18 (130) ethylene bridged hybrid (BEH) beads (Waters Corporation). Peptides were eluted from the C18 column into the mass spectrometer using a linear gradient (5 to 80%) of ACN (acetonitrile) at a flow rate of 375 μl/min for 1 hour. The buffers used to create the ACN gradient were as follows: buffer A (98% H_2_O, 2% ACN, and 0.1% formic acid) and buffer B (100% ACN and 0.1% formic acid). Mass spectrometer parameters were as follows: An MS1 survey scan using the Orbitrap detector {mass range [mass/charge ratio (*m/z*)]: 400 to 1500 (using quadrupole isolation), 120,000 resolution setting, spray voltage of 2200 V, ion transfer tube temperature of 275°C, automatic gain control (AGC) target of 400,000, and maximum injection time of 50 ms} was followed by data-dependent scans (top speed for most intense ions, with charge state set only to include +2 to 5 ions, and 5-s exclusion time), while selecting ions with minimal intensities of 50,000 in which the collision event was carried out in the high-energy collision cell [higher-energy C-trap dissociation (HCD) collision energy of 30%], and the fragment masses were analyzed in the ion trap mass analyzer (with ion trap scan rate of turbo, first mass *m/z* was 100, AGC target 5000, and maximum injection time of 35 ms). Protein identification and SILAC quantification were carried out using Peaks Studio 8.5 (Bioinformatics Solutions Inc.) For modifications, l-arginine-(^13^C_6_,^15^N_4_) was set as a variable modification and peptides with this modification were used for SILAC quantification. Regulon assignments were made in accordance with those found on the SubtiWiki databases (http://subtiwiki.uni-goettingen.de/) (*23*). Genes expressed as part of the SigA, SigH, and/or Spo0A regulons were considered vegetative.

### Statistical and computational methods

#### Automated programs

All codes for automated regulon assignment and for the automated quantification of fluorescence ratios and phase brightness can be accessed and are described at https://github.com/PoglianoLab/MetabolicDifferentiation.

#### Statistical analysis

Number of trials and error bars are described in figure legends.

## SUPPLEMENTARY MATERIALS

Supplementary material for this article is available at http://advances.sciencemag.org/cgi/content/full/7/4/eabd6385/DC1

## Appendix

View/request a protocol for this paper from *Bio-protocol*.
